# Gut microbiota-derived 4-hydroxyphenylacetic acid from resveratrol supplementation prevents obesity through SIRT1 signaling activation

**DOI:** 10.1080/19490976.2024.2446391

**Published:** 2024-12-26

**Authors:** Pan Wang, Ruiqi Wang, Wenting Zhao, Yuanyuan Zhao, Dan Wang, Shuang Zhao, Zhiwen Ge, Yue Ma, Xiaoyan Zhao

**Affiliations:** a Institute of Agri-Food Processing and Nutrition, Beijing Academy of Agriculture and Forestry Sciences, Beijing, China; b Beijing Key Laboratory of Agricultural Products of Fruits and Vegetables Preservation and Processing, Key Laboratory of Vegetable Postharvest Processing, Ministry of Agriculture and Rural Affairs, Beijing, China

**Keywords:** Resveratrol, 4-hydroxyphenylacetic acid, gut microbiota, obesity, SIRT1

## Abstract

Resveratrol (RSV), a natural polyphenol, has been suggested to influence glucose and lipid metabolism. However, the underlying molecular mechanism of its action remains largely unknown due to its multiple biological targets and low bioavailability. In this study, we demonstrate that RSV supplementation ameliorates high-fat-diet (HFD)-induced gut microbiota dysbiosis, enhancing the abundance of anti-obesity bacterial strains such as *Akkermansia, Bacteroides* and *Blautia*. The critical role of gut microbiota in RSV-mediated anti-obesity effects was confirmed through antibiotic-induced microbiome depletion and fecal microbiota transplantation (FMT), which showed that RSV treatment effectively mitigates body weight, histopathological damage, glucose dysregulation and systematic inflammation associated with HFD. Metabolomics analysis revealed that RSV supplementation significantly increases the levels of the gut microbial flavonoid catabolite 4-hydroxyphenylacetic acid (4-HPA). Notably, 4-HPA was sufficient to reverse obesity and glucose intolerance in HFD-fed mice. Mechanistically,4-HPA treatment markedly regulates SIRT1 signaling pathways and induces the expression of beige fat and thermogenesis-specific markers in white adipose tissue (WAT). These beneficial effects of 4-HPA are partially abolished by EX527, a known SIRT1 inhibitor. Collectively, our findings indicate that RSV improve obesity through a gut microbiota-derived 4-HPA-SIRT1 axis, highlighting gut microbiota metabolites as a promising target for obesity prevention.

## GRAPHICAL ABSTRACT

**Figure:**
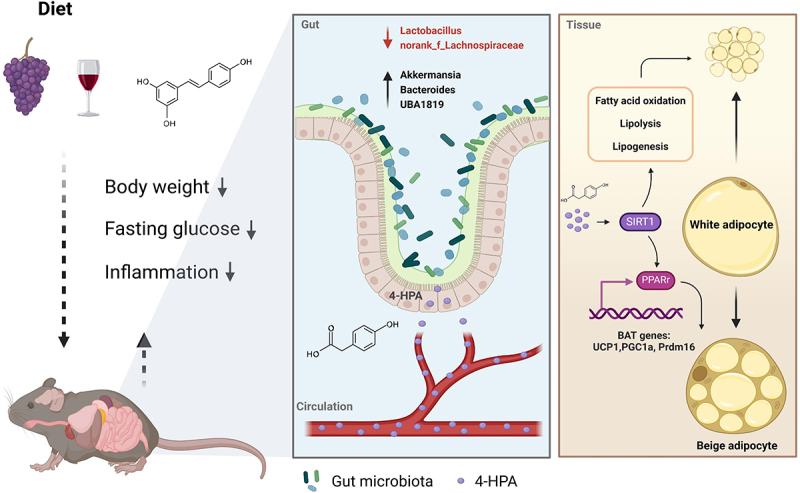

## 1. Introduction

Obesity has become a significant global health issue, with the World Health Organization reporting more than a twofold increase in adult obesity rates and a fourfold rise in adolescent obesity since 1990.^1^ This complex metabolic disorder, characterized by excessive fat accumulation and disrupted energy homeostasis, is influenced by genetic, environmental, and lifestyle choices.^2^ Obesity is associated with numerous chronic diseases, including nonalcoholic fatty liver disease, type 2 diabetes, cardiovascular diseases and certain cancers.^3^ Given the critical role of adipose tissue in energy regulation, research has concentrated on activating adaptive thermogenesis in both white adipose tissue (WAT) and brown adipose tissue (BAT). Despite various treatments such as calorie restriction, physical activity and pharmacotherapy, these approaches often exhibit side effects, limited efficacy and eventual relapse.^4^ Thus, there is an urgent need for novel, effective strategies to induce browning in WAT and enhance BAT activity to prevent and treat obesity.

Mammalian Sirtuin 1 (SIRT1), a NAD^+^-dependent deacetylase enzyme, is pivotal in regulating energy homeostasis and adipocyte browning.^5^ Evidence suggests a significant role of SIRT1 in regulating thermogenesis genes and its levels are inversely correlated with body mass index (BMI) and adipose tissue macrophage infiltration.^6,7^ Study have shown that SIRT1 downregulation occurs in obesity, while its overexpression protects against obesity-related outcomes by enhancing adipose tissue lipolysis, inducing WAT browning, and activating BAT thermogenesis.^8–11^ Given the rising prevalence of metabolic disorders, compounds that activate SIRT1 could mitigate metabolic damage.

Resveratrol (RSV), a natural polyphenolic compound found in red wine, grapes and jackfruit, has shown beneficial effects on cardiovascular disease and metabolic disease, including obesity, diabetes and fatty liver disease.^12^ *In vitro* studies using fluorophore-targeted substrate have identified RSV as an activator of SIRT1 deacetylase activity.^13^ However, recent evidence challenges the notion that RSV directly activates SIRT1. It has been demonstrated that RSV’s activation of SIRT1 is dependent on the presence of a covalently attached fluorophore in the substrate, suggesting that RSV may exert it’s *in vivo* effects through mechanisms other than direct SIRT1 activation. Additionally, RSV exhibits low bioavailability, with free RSV levels in plasma constituting less than 2% of the administered dose and being virtually undetectable in tissues other than intestinal.^14^ RSV remains localized in the intestine for up to 24 h following oral administration in the mouse model.^15^ Consequently, dietary RSV may primarily function within the gut, where its concentration is highest, potentially by interacting with gut microbiota and their metabolites. Previous studies have shown that dietary RSV profoundly influences gut microbiota composition and metabolic function.^16,17^ Notably, nutrients that are not fully metabolized by the host are processed by gut microbiota into beneficial metabolites, such as short-chain fatty acids and bile acids,^18^ which then enter circulation and mediate interaction between the host and microbiota. These findings suggest that gut microbiota may help overcome RSV’s poor bioavailability by converting it into active metabolites.^19,20^ However, the specific role of microbiota-derived metabolites in modulating the effects of RSV on lipid metabolism in HFD-fed mice, and the underlying mechanisms, have yet to be fully elucidated.

In this study, we reveled that RSV significantly improves gut microbiota dysbiosis in HFD-fed mice. Through pharmacological and genetic approaches, we identified and confirmed RSV’s role and mechanism in mitigating obesity. Antibiotic treatment and fecal microbiota transplantation (FMT) validate that RSV’s protective effects are modulated by the gut microbiota. Metabolomic analysis identified 4-hydroxyphenylacetic acid (4-HPA), a gut microbiota-derived metabolites of RSV, as mediating RSV’s beneficial effects on HFD-induced obesity through SIRT1 signaling. Our findings provide new insights into the mechanisms by which RSV protects against obesity and highlight the potential of 4-HPA as a postbiotic for regulating HFD-induced obesity.

## 2. Materials and methods

### 2.1. Animal experimental design

All animal experiments were conducted in accordance with the guidelines set forth by the Biomedical Ethical Committees of Peking University (Beijing, China), under approval number LA2021305. Six-week-old male C57BL/6J mice (Beijing Vital River Laboratory Animal Technology Co., Ltd., Beijing, China) were housed four per cage in a standard specific pathogen-free (SPF) environment with 12-hour light/dark cycle, maintained at 20–22°C and 45 ± 5% humidity.

#### 2.1.1. Animal experiment 1: RSV and antibiotic treatments

After a one-week acclimation period, 24 mice were randomly divided into 3 groups for a duration of 16 weeks: Normal Control Diet (NCD, 10% dietary fat, *n* = 8, Cat#D12450B) group; High-Fat Diet (HFD, 60% dietary fat, *n* = 8, Cat#D12492), and HFD with Resveratrol (HFD+RSV, 300 mg/kg/day by gavage, *n* = 8). The NCD and HFD groups served as controls and received an equivalent volume of normal saline. The RSV (Sigma-Aldrich, USA) dose was based on previous studies.^16,17^ The dietary ingredients and energy densities are detailed in Supplementary Table S1 (Research Diet, New Brunswick, NJ, USA). Body weight (BW) and food intake were recorded biweekly. Mean energy intake was calculated based on the mean weight of dietary intake (per day per mouse) and the energy density of diets. At the end of the experiment, all mice were euthanized under ether anesthesia via cervical dislocation. Blood, tissue and fecal samples were collected for further analysis.

In a separate study, to assess RSV’s effects on obesity in the absence of gut microbiota, mice were pretreated with a combination antibiotics (ampicillin, 1 g/L; metronidazole, 1 g/L; vancomycin, 0.5 g/L; neomycin, 0.5 g/L; ciprofloxacin 0.5 g/L) (Sigma-Aldrich, USA) to deplete the gut microbiota. Following one week of acclimation, these microbiota-depleted mice were administered either HFD (anti-HFD) or HFD with RSV (anti-HFDR) for 16 weeks (*n* = 8 per group). Antibiotics were initially administered via oral gavage (200 μL daily) for 1 week and then provided ad libitum in drinking water.

#### 2.1.2. Animal experiment 2: fecal microbiota transplants (FMT)

Fecal microbiota transplants following a protocol with minor modifications from previous studies [23]. Six-week-old male recipient HFD-fed mice (*n* = 8 per group) were initially treated with antibiotics in their drinking water for 14 days, after which the water was replaced with regular water. Recipient mice then received daily fecal transplants (200 μL) via oral gavage. Briefly, donor mice were fed either HFD (HFD-HFD) or HFDR (HFD-HFDR) for 16 weeks (*n* = 9 per group). Fresh fecal samples were collected, mixed (200 mg stools per sample) with sterile saline, vortexed and centrifuged. The supernatant was prepared immediately before transplantation. After 16 weeks, recipients were euthanized, and tissues were collected.

#### 2.1.3. Animal experiment 3: treatment with 4-HPA derived from RSV

To evaluate the effects of the gut microbiota-derived metabolite 4-hydroxyphenylacetic acid (4-HPA) from RSV on obesity, 24 mice were divided into three groups for 16 weeks: NCD (10% dietary fat, *n* = 8), HFD (60% dietary fat, *n* = 8), and HFD + 4-HPA (HFD4A, 30 mg/kg/day by gavage, *n* = 8). NCD and HFD groups received an equivalent volume of normal saline. At the end of the experiment, mice were euthanized under ether anesthesia by cervical dislocation, and blood, tissue, and fecal samples were collected.

#### 2.1.4. Animal experiment 4: treatment with SRIT1 antagonist EX527

To investigate the role of SIRT1 in mediating the effects of 4-HPA, 32 mice were divided into four groups for 16 weeks: HFD (60% dietary fat, *n* = 8), HFD + 4-HPA (30 mg/kg/day by gavage, *n* = 8), HFD+EX527 (10 mg/kg/day by gavage, *n* = 8), and HFD + 4-HPA+EX527 (*n* = 8). At the end of the experiment, mice were euthanized under ether anesthesia by cervical dislocation, and blood, tissue, and fecal samples were collected.

### 2.2. Inflammatory cytokine analysis

Serum levels of inflammatory cytokines – interleukin-1β (IL-1β), interleukin-6 (IL-6), interleukin-10 (IL-10), and tumor necrosis factor-α (TNF-α) – were measured using enzyme-linked immunosorbent assay (ELISA) kits (Nanjing Jiancheng Bioengineering Institute Co., Ltd., Nanjing, China), following the manufacturer’s instructions.

### 2.3. Histological analysis

Epididymal and interscapular adipose tissues were fixed in 4% paraformaldehyde, dehydrated, cleared, paraffin-embedded, and sectioned at 5 µm. Sections were stained with hematoxylin and eosin (H&E) (Sigma-Aldrich, St. Louis, MO, USA) as previously reported.^18^

### 2.4. Glucose (GTT) and insulin tolerance (ITT) tests

At week 12, mice were fasted for 16 hours prior to glucose administration (1.0 g/kg BW). Blood glucose levels was measured at 0, 15, 30, 60, 90 and 120 minutes using a glucometer (Accu-chek, Roche). In addition, at week 13, following a 6-hour fast, mice received an intraperitoneal insulin injection (0.75 UI/kg; Novo Nordisk), and blood glucose levels were measured similarly.

### 2.5. Gut microbiota analysis

Genomic DNA was extracted from fecal samples (*n* = 7 per group) using the QiAamp® Fast DNA Stool Mini Kit (Qiagen, Hilden, Germany). Sequencing targeted the V3-V4 regions using universal primers 338F and 806 R on the Illumina MiSeq platform (Majorbio Bio-Pharm Technology Co., Ltd., Shanghai, China). Detailed sequencing methods are outlined in the Supplemental Methods.

### 2.6. Non-targeted metabolomics

Gas chromatography-mass spectrometry (GC-MS) analysis were performed by ProfLeader Biotech Co., Ltd. (Shanghai, China). Fecal samples were homogenized in 20-fold-volume 50% cold methanol containing 5 μg/mL L-norleucine. The mixture was vortexed for 1 min and stood for 30 min at −20°C. After centrifugation at 14,000 g for 15 min at 4°C, 100 μL supernatants and 5 μL of 50 μg/mL L-norvaline were evaporated to dryness under nitrogen stream. The residue was reconstituted in 30 μL of 20 mg/mL methoxyamine hydrochloride in pyridine (containing 5 μg/mL n-alkanes standards), and the resulting mixture was incubated at 37°C for 90 min. A 30 μL of BSTFA (with 1% TMCS) was added into the mixture and derivatized at 70°C for 60 min prior to GC-MS metabolomics analysis. Instrumental analysis was performed on an Agilent 7890A/5975C GC-MS system (Agilent Technologies Inc., CA, USA). An OPTIMA® 5 MS Accent fused-silica capillary column (30 m × 0.25 mm × 0.25 μm; MACHEREY-NAGEL, Düren, GERMAN) was utilized to separate the derivatives. Helium (>99.999%) was used as a carrier gas at a constant flow rate of 1 mL/min through the column.

### 2.7. Targeted quantification of aromatic metabolites

A 50 mg (±5 mg) aliquot of frozen feces was homogenized in 500 μL cold water containing an internal standard (10 μg/mL of L-norvaline). After centrifugation at 14,000 g and 4°C for 15 min, the supernatant was collected. The extraction process was repeated, and the supernatants were combined. The combined supernatant (500 μL) was then evaporated to dryness under a nitrogen stream. The resulting dry residue was reconstituted in 30 μL of pyridine and 30 μL of BSTFA (containing 1% TMCS) and derivatized at 70°C for 60 minutes prior to GC-MS analysis. For quantification, a mixed standard solution containing 20 μg/mL of 4-hydroxyphenylacetic acid, 4-hydroxyphenylpropionic acid, 3-hydroxyphenylpropionic acid, 3-hydroxybenzoic acid and resveratrol, was prepared in water and serially diluted to concentrations ranging from 0.02 ~ 10 μg/mL, respectively.

### 2.8. Quantitative real-time PCR (qRT-PCR) analysis

Total RNA was extracted from liver and ileum tissues using Trizol (Thermo Fisher Scientific, Haverhill, MA, USA) and subsequently purified with the RNeasy Mini Kit (Qiagen, Frederick, MD, USA). Reverse transcription polymerase chain reaction (RT-PCR) was conducted with SYBR Green on the LightCycler 480 Real-Time PCR system (Roche Diagnostics). The primers used are detailed in Supplementary Table S2 Gene expression levels were normalized to GAPDH. Relative quantification was performed using the 2−ΔΔCt method.

Total genomic DNA of fecal and cultured fecal samples was extracted using a QIAamp-DNA Stool Mini Kit (Qiagen, Hilden, Germany). The integrity of the extracted DNA was examined by electrophoresis in 1% (wt/vol) agarose gels. The template DNA (0.5 μl) were mixed with 1 μl of the target primers, 5 μl of SYBR Green I Master Mix (Roche Diagnostics, Basel, Switzerland) and 3.5 μl of water. PCRs were performed in triplicates on a LightCycler 480 Real-Time PCR system using the following PCR program: initial preincubation at 95°C for 30 s, 40 PCR cycles of 95°C for 5 s, Tm for 30 s, 72°C for 30 s, followed by a melting curve. For bacteria quantification, standard curves were prepared using continuous dilution of purified and quantified PCR products generated from the bacterial genomic DNA.

### 2.9. Western blot analysis

Frozen mouse tissues were homogenized in lysis buffer with the Complete Protease Inhibitor Cocktail (ThermoFisher Scientific, Waltham, MA) to extract total tissue protein. Protein concentrations were measured using the bicinchoninic acid (BCA) Protein Assay Kit (Beyotime, Shanghai, China), with bovine serum albumin serving as the standard. For Western blot analysis, 40 μg of protein aliquots were denatured by boiling in Tris-Glycine SDS Sample Buffer (Bio-Rad) and separated by SDS-PAGE. The proteins were then transferred onto nitrocellulose membranes (Bio-Rad) via electroblotting. Membranes were blocked with Pierce Protein-free Tween 20 Blocking Buffer (ThermoFisher Scientific) for 1 hour and incubated with specific primary antibodies (listed in Supplementary Table S3). β-Actin (Sigma-Aldrich) served was used as loading control. Following primary antibody incubation, membranes were probed with horseradish peroxidase-conjugated secondary antibody (Jackson Immunoresearch Laboratories Inc., West Grove, PA) diluted 1:5000 for 30 minutes. Proteins detection was performed using the Super Signal West Femto Chemiluminescent Substrate (Pierce Chemical Co., New York, NY).

### 2.10. Statistical analysis

The data were analyzed using GraphPad Prism (GraphPad Software 5.0) and presented as mean ± SEM. For comparisons involving more than two groups, one-way ANOVA followed by Tukey’s post hoc test was employed. Two-group comparisons were performed using t-tests. Next-generation sequencing data were analyzed with Tukey’s Honestly Significant Difference post hoc tests. Statistical significance was set at *p* < 0.05. Differences between groups are indicated by distinct superscript letters in the figures. The graphical abstract was designed using BioRender.

## 3. Results

### 3.1. RSV reverses gut dysbiosis in HFD-fed mice

**Figure 1.**
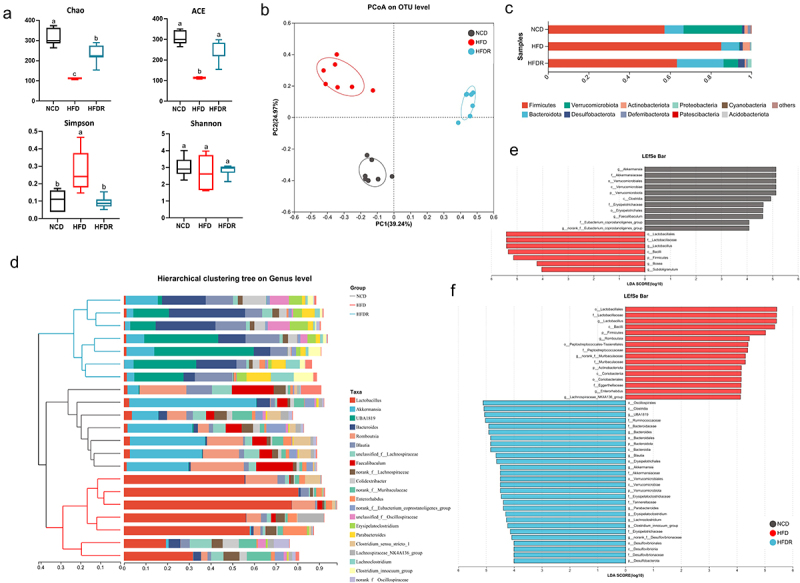
RSV ameliorated gut microbiota dysbiosis of HFD mice. (a) the α-diversity including Chao, ACE, Simpson and Shannon index of gut microbiota among different groups. (b) Weighted UniFrac PCoA analysis. (c) Community bar plot analysis at the phylum level. (d) The dendrogram clustering and the bacterial taxonomic profiling in 20 main genera of the gut microbiota. (e, f) linear discriminant analysis (LDA) effect size (LEfSe) was calculated to explore the taxa within the lowest taxonomic level possible that more strongly discriminate between the gut microbiota of NCD vs HFD and HFD vs HFDR. Graph bars in a marked with different letters on top represent statistically significant results (*p* < 0.05).

Previous research, including our own, has established that RSV supplementation can mitigate HFD-induced obesity. However, the precise mechanism underlying this effect remains largely unclear, partly due to RSV’s poor bioavailability. Considering the interplay between gut microbiota, obesity and dietary nutrition, we investigated whether RSV could counteract HFD-induced gut microbiota dysbiosis. We assessed the gut microbiota composition using 16S rRNA gene amplicon sequencing. For α-diversity analysis, HFD-fed mice exhibited lower richness of gut microbiota than NCD-fed mice, as indicated by an elevated Shannon index and reduced ACE and Chao values (Figure 1(a)). Upon RSV supplementation, there was a significant increase in α-diversity, particularly in the Shannon, ACE and Chao index. To further elucidate the structural changes in the gut microbial community, non-metric multidimensional scaling (NMDS) and UniFrac-based principal coordinates analysis (PCoA) were employed at the genus level. These results demonstrated significant differences in microbial communities among NCD, HFD and HFDR groups (Figure 1(b), Supplementary Figure S1A).

To elucidate specific changes in gut microbiota composition, we analyzed the relative abundance of the predominant taxa in different groups. At the phylum level, HFD increased the abundance of *Firmicutes* and *Actinobacteriota*, while decreasing *Verrucomicrobiota*. RSV administration contracted these HFD-induced changes (Figure 1(c)). Additionally, RSV treatment notably increased the abundance of *Bacteroidetes* in HFD-fed mice (Figure 1(c), Supplementary Figure S1B, C). were the dominant phyla in the three groups. At the genus level, RSV prevented the HFD-induced reduction in *Akkermansia* and *Blautia*, and decreased the relative abundance of *Lactobacillus*, which accounted for most of the reduction in *Firmicutes* observed in the RSV-treated group compared to the HFD group (Figure 1(d) and Supplementary Figure S1D, E). LEfSe analysis identified *Akkermansia* and *Faecalibaculum* as key discriminators of fecal bacterial communities between NCD and HFD-fed mice. HFD mice exhibited higher abundance of *Lactobacillus*^21^, *Enterorhabdus (Coriobacteriaceae)*^22^, *Erysipelotrichaceae_UCG-003*^23^ and *Lachnospiraceae_NK4A136_group*,^24^ genera known to be positively correlated with obesity and related disorders (Figure 1(e, f)). In contrast, the HFDR group showed significantly increased abundance of *UBA1819, Bacteroides, Akkermansia* and *Blautia*. In summary, these findings demonstrate that RSV administration protects against HFD-induced gut microbiota dysbiosis, which may contribute to the alleviation of obesity.

### 3.2. Gut microbiota mediates the anti-obesity function of RSV in HFD-fed mice

**Figure 2.**
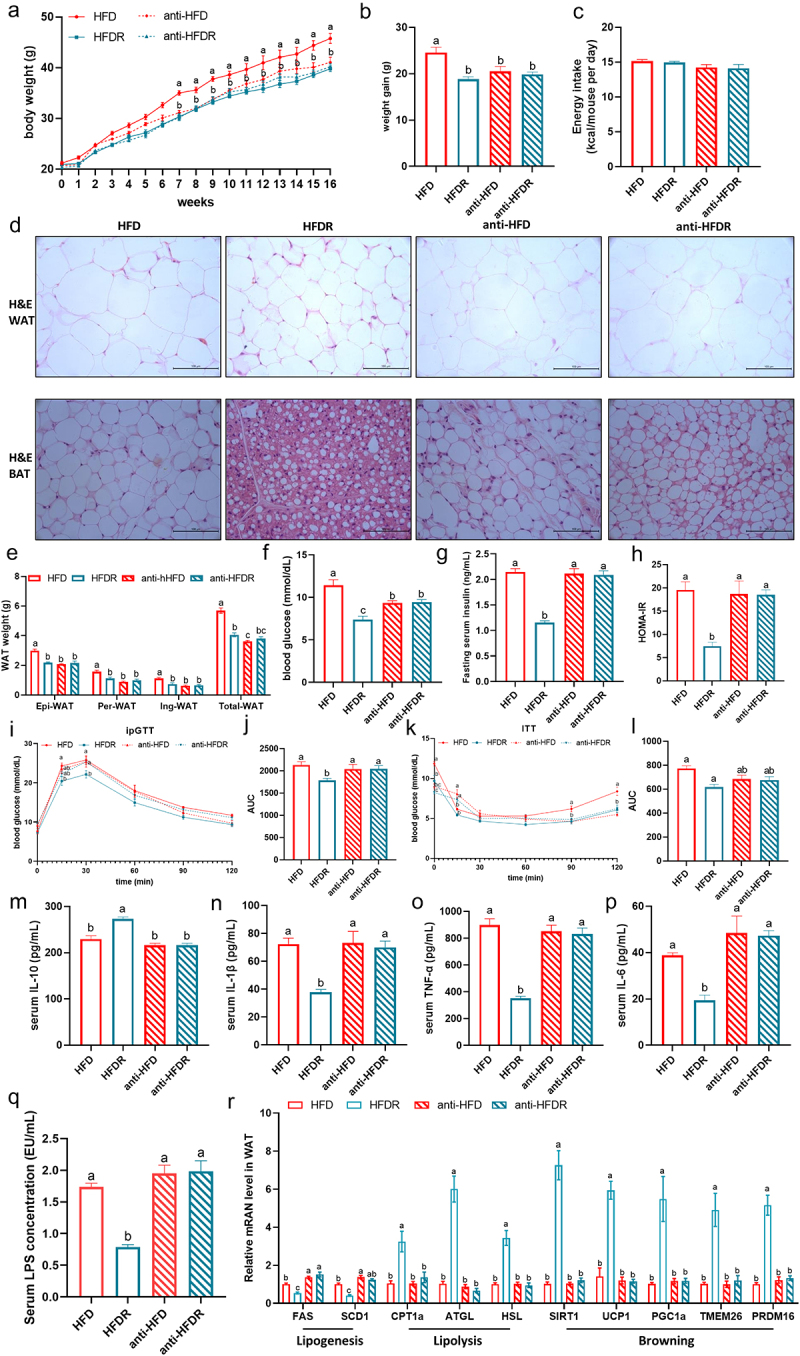
Gut microbiota is involved in obesity prevention by RSV. mice treated with an antibiotic cocktail were fed with HFD or HFDR for 16 weeks (anti-hfd, anti-hfdr, respectively), after which the following analyses were performed. (a) Body weight. (b) Body weight gain. (c) Energy intake. (d) H&E staining of white adipose and brown adipose tissue sections (scale: 100 μm). (e) WAT weight. (f) blood glucose. (g) Fasting serum insulin. (h) HOMA-ir. (i) Intraperitoneal glucose tolerance test (ipGTT) and (j) the calculated AUC. (k) Insulin tolerance test (ITT) and (l) the calculated AUC for ITT. (m) Serum IL-10 level. (n) Serum IL-1β level. (o) Serum tnf-α level. (p) Serum IL-6 level. (q) Serum LPS level. (r) The mRNA expression of lipid metabolism in WAT. Values are expressed as the mean±sem. Different letters (a, b and c) mean significant difference in different treatment groups (*p* < 0.05). WAT white adipose tissue, epi-wat epididymal adipose tissue, per-wat perirenal adipose tissue, ing-wat interscapular adipose tissue, total WAT = epi-WAT+Per-WAT+Ing-wat.

To determine whether the anti-obesity effects of RSV in the HFD-fed mice are mediated by gut microbiota, we administered an antibiotic cocktail to deplete gut microbiota in HFD and HFDR group. The content of fecal-extracted DNA was almost 100-fold lower in the antibiotic-treated mice than in the mice without antibiotic treatment (Supplementary Table S4), suggesting a remarkable depletion of gut bacteria. As shown in Figure 2(a-b), RSV treatment significantly reduced body weight in the HFDR groups. However, antibiotic treatment led to no significant difference in body weight between the anti-HFD and anti-HFDR groups, indicating that RSV lost its efficacy in reducing HFD-induced body weight when gut microbiota was depleted. No significance difference was observed in energy intake among the HFD, HFDR, anti-HFD and anti-HFDR (Figure 2(c)). In line with the reduction in WAT weight, histological analysis displayed that large adipocytes were most abundant in the WAT and BAT of HFD mice, whereas adipocyte size was significantly reduced with RSV treatment (Figure 2(d, e)). Next, we investigated the possible actions of RSV administration on the metabolic parameters including glucose homeostasis and insulin resistance. RSV group had a greater ability to lower fasting blood glucose levels, insulin concentrations and HOMA-IR index (Figure 2(f-h)). Results of the OGTT and ITT, and the corresponding area under curve values, further confirmed that RSV administration notably ameliorated the impaired glucose tolerance and insulin resistance in HFD-fed mice (Figure 2(i-l)). Furthermore, RSV supplementation significantly decreased the levels of IL-1β, TNF-α, IL-6 and LPS and increased the content of IL-10 in the serum of the HFD-fed mice, suggesting that endotoxemia and systematic inflammation in obese mice were alleviated by RSV supplementation (Figure 2(m-q)). However, RSV failed to prevent HFD-induced obesity-related parameters, including fat mass (Figure 2(d, e)), glucose intolerance (Figure 2(f-l)), systematic inflammation and serum LPS (Figure 2(m-q)), when gut microbiota was depleted. RSV also did not regulate lipogenesis genes (FAS and SCD1), lipolysis genes (CPT1α, ATGL and HSL) or browning related genes (SIRT1, UCP1, PGC1α, TMEM26 and PRDM16) in Epi-WAT (Figure 2(r)). These findings suggest that the protective effects of RSV against are likely associated with its modulation of gut microbiota.

**Figure 3.**
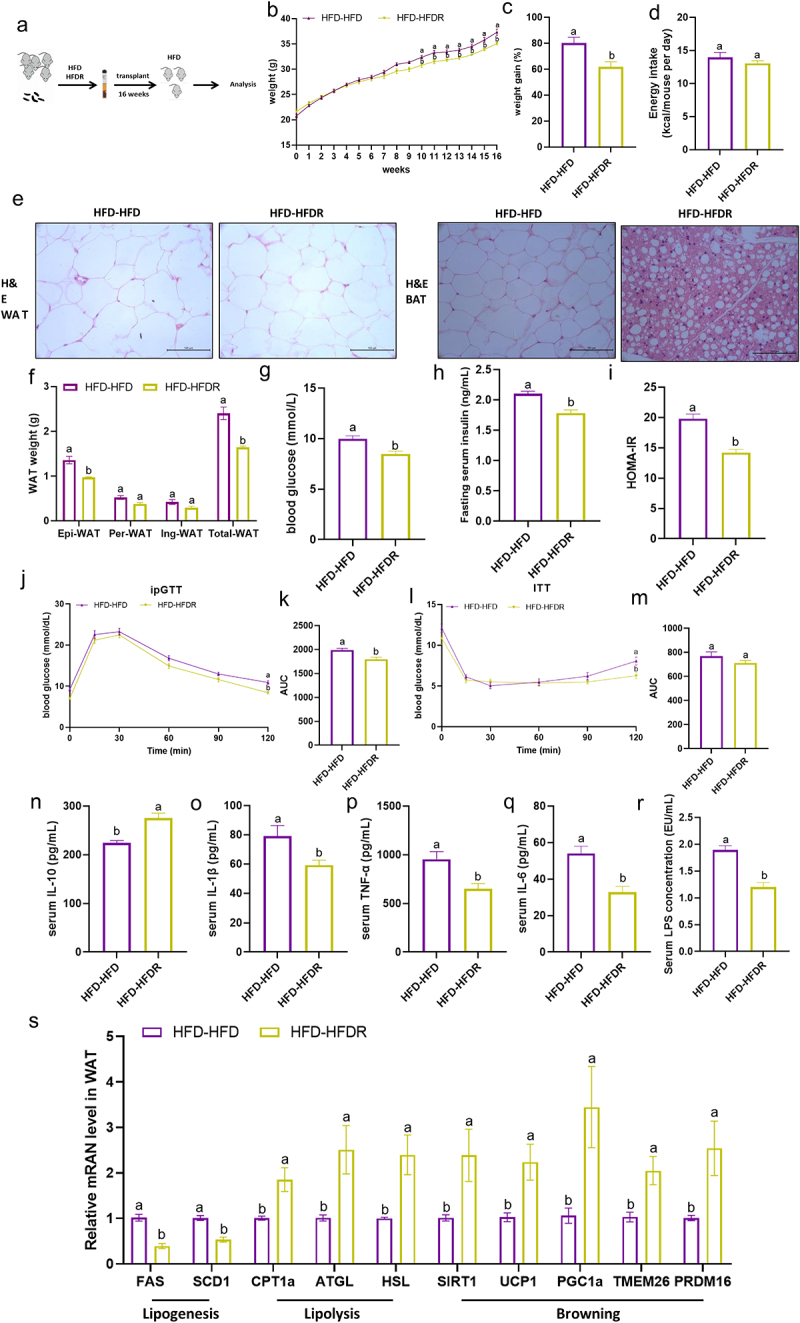
The beneficial effect of RSV on hfd-fed mice were transferred by FMT. hfd-fed mice were divided into the following two groups (*n* = 8–10) on 16 weeks: HFD-HFD group (administrated daily with microbiota from the HFD group), HFD-HFDR group (administrated daily with microbiota from the HFDR group). (a) Study design of FMT in hfd-fed mice. (b) Body weight. (c) Body weight gain. (d) Energy intake. (e) H&E staining of white adipose and brown adipose tissue sections (scale: 100 μm); (f) WAT weight. (g) blood glucose. (h) Fasting serum insulin. (i) HOMA-ir. (j) Intraperitoneal glucose tolerance test (ipGTT) and (k) the calculated AUC. (l) Insulin tolerance test (ITT) and (m) the calculated AUC for ITT. (n) Serum IL-10 level. (o) Serum IL-1β level. (p) Serum tnf-α level. (q) Serum IL-6 level. (r) Serum LPS level. (s) The mRNA expression of lipid metabolism in WAT. Values are expressed as the mean ± sem. Different letters (a, b and c) mean significant difference in different treatment groups (*p* < 0.05). WAT white adipose tissue, epi-wat epididymal adipose tissue, per-wat perirenal adipose tissue, ing-wat interscapular adipose tissue, total WAT = epi-WAT+Per-WAT+Ing-wat.

To further investigate whether RSV-induced gut microbiota could alleviate obesity, we performed FMT in HFD-fed mice (Figure 3(a)). As expected, mice receiving microbiota from HFDR-treated donors (HFD-HFDR) exhibited improved metabolic profiles compared to those receiving microbiota from HFD-fed mice (HFD-HFD), including reductions in body weight, weight gain, WAT weight and adipocyte size (Figure 3(b-f)). No significant differences in mean energy intake between HFD-HFD and HFD-HFDR groups (Figure 3(d)). Additionally, fasting blood glucose, insulin levels, HOMA-IR index, and glucose and insulin intolerance were significantly improved in the HFD-HFDR group (Figure 3(g-m)). Consistent with RSV supplementation results, FMT from HFDR mice also significantly reduced systematic inflammation and serum LPS levels (Figure 3(n-r)). The mRNA expression of lipogenic (FAS and SCD1), fatty acid oxidation (CPT1α, ATGL and HSL) and browning genes (SIRT1, UCP1, PGC1α, TMEM26 and PRDM16) were also improved in WAT following transfer of feces derived from HFDR-treated mice (Figure 3(s)). In summary, the beneficial effects of RSV are transferable via gut microbiota.

### 3.3. RSV significantly enriches gut microbiota-derived aromatic metabolites

**Figure 4.**
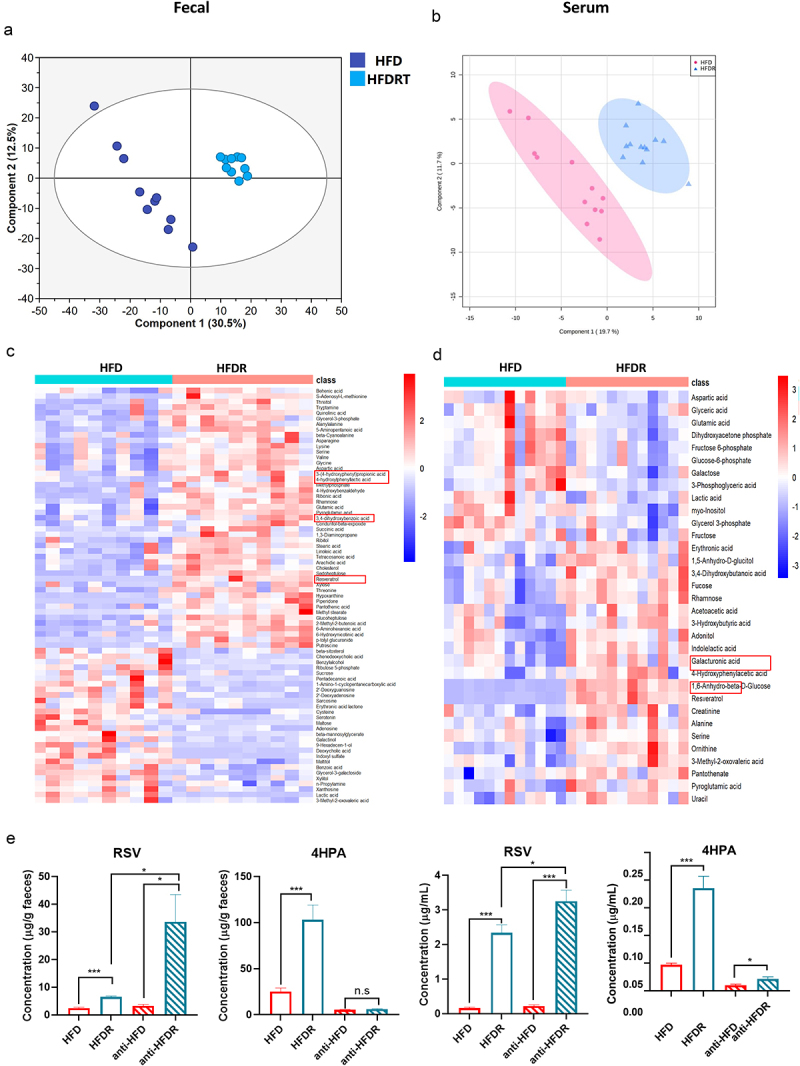
RSV alters metabolic profiling and enrich microbiota-derived aromatic acid. mice were fed with HFD or HFDR (300 mg/kg/day) for 16 weeks. (a, b) Principal components analysis (PCA) score plots for discriminating the fecal and serum metabolome from HFD and HFDR group. (c, d) Heatmap of the differential metabolites in fecal and serum that were altered by RSV. (e, f) concentration of RSV and 4-HPA in stools and serum of HFD and HFDR group with or without antibiotics. Data are pooled from three independent experiments. Values are expressed as the mean±sem. Different letters (a, b and c) mean significant difference in different treatment groups (*p* < 0.05).

The gut microbiota plays a crucial role in metabolizing dietary nutrients into various metabolites that mediate host-microbiota interactions. We hypothesized that RSV prevent obesity in HFD-fed mice through metabolites generated by microbial fermentation. To investigate metabolic changes resulting from RSV-induced microbiota remodeling, we conducted untargeted metabolome profiling of fecal and serum samples using gas chromatography coupled to mass spectrometry (GC-MS). A total of 963 and 159 metabolites were identified in fecal and serum samples, respectively (Supplementary Table S5, S6), with 75 and 31 metabolites differentially enriched between the HFD and HFDR group (Supplementary Figure S2A, B). Principal components analysis (PCA) and partial least squares discrimination analysis (PLS-DA) demonstrated distinct clustering of HFD and HFDR groups (Figure 4(a, b), Supplementary Figure S2C, D). In the fecal metabolome, 47 metabolites were upregulated and 28 metabolites were downregulated in the HFDR group relative to the HFD group. In serum samples, 21 metabolites were upregulated and 12 were downregulated in the HFDR compared to the HFD group. Among these fecal metabolites, RSV treatment notably increased levels of 3,4-dihydroxybenzoic acid, 3-(4-hydroxyphenyl) propionic acid and 4-hydroxyphenylacetic acid (4-HPA), all of which share aromatic structures (Figure 4c, d). Importantly, 4-HPA was the sole metabolite present in both fecal and serum samples, exhibiting a similar variation pattern following RSV intervention. The metabolomic pathway analysis revealed that RSV treatment significantly enriched pathway related to amino acid metabolism, including glycine, serine and threonine metabolism, arginine and proline metabolism and tyrosine metabolism (Supplementary Figure S2E, F, Supplementary Table S7, S8). We further analyzed the correlations between obesity-related parameters (weight, weight gain, glucose, insulin and inflammation factor) and changes in differential metabolites using Spearman correlation analysis (Supplementary Figure S2G, H). Aromatic acid metabolites, specially 3,4-dihydroxybenzoic acid, 3-(4-hydroxyphenyl) propionic acid and 4-hydroxyphenylacetic acid (4-HPA), were upregulated in RSV-treated mice and negatively correlated with obesity features.

To assess whether the RSV-induced enrichment of 4-HPA is dependent on gut microbiota, we administered broad-spectrum antibiotics to both HFD and HFDR-fed mice to deplete gut microbiota and subsequently measured 4-HPA and RSV levels using targeted analysis. Consistent with the metabolomic findings, RSV-treated mice exhibited significantly higher levels of 4-HPA and RSV in both fecal and serum samples compared to HFD controls. Notably, RSV levels were elevated in the feces and serum of antibiotic-treated HFDR mice compared to HFDR mice, suggesting that gut microbiota contributes to RSV metabolism. Moreover, antibiotic treatment markedly reduced 4-HPA levels in fecal and serum samples from both HFD and HFDR groups, with no significant difference between antibiotic-treated HFD and HFDR groups, indicating that RSV enhances 4-HPA levels in a microbiota-dependent manner. Consequently, we selected 4-HPA as a candidate molecule to test the hypothesis that gut microbiota-derived metabolites of RSV are sufficient to mitigate HFD-induced metabolic disorders.

### 3.4. 4-HPA exert anti-obesity effects in HFD-fed mice

**Figure 5.**
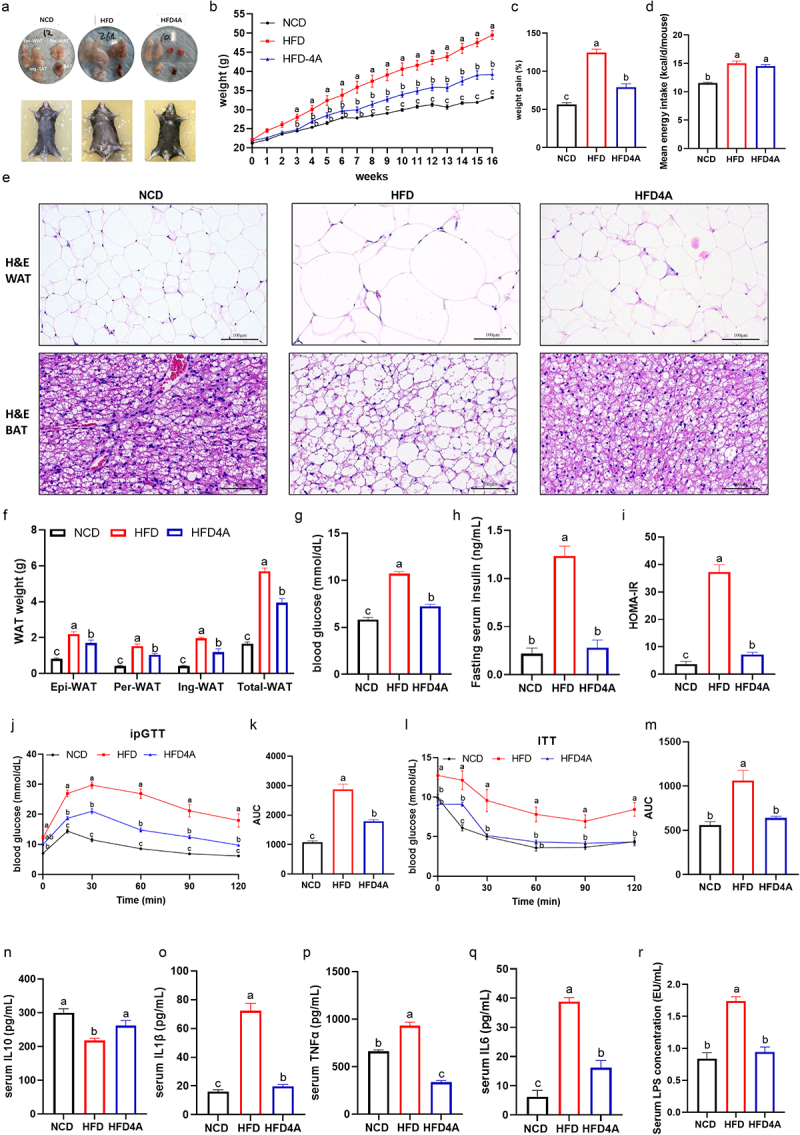
4-HPA is sufficient to reverse hfd-induced obesity. mice were fed with NCD, HFD or HFD4A (30 mg/kg/day) for 16 weeks. (a) Whole-body images of mice in different groups. (b) Body weight. (c) Body weight gain. (d) Energy intake. (e) H&E staining of white adipose and brown adipose tissue sections (scale: 100 μm); (f) WAT weight. (g) blood glucose. (h) Fasting serum insulin. (i) HOMA-ir. (j) Intraperitoneal glucose tolerance test (ipGTT) and (k) the calculated AUC. (l) Insulin tolerance test (ITT) and (m) the calculated AUC for ITT. (n) Serum IL-10 level. (o) Serum IL-1β level. (p) Serum tnf-α level. (q) Serum IL-6 level. (r) Serum LPS level. Values are expressed as the mean±sem. Different letters (a, b and c) mean significant difference in different treatment groups (*p* < 0.05). WAT white adipose tissue, epi-wat epididymal adipose tissue, per-wat perirenal adipose tissue, ing-wat interscapular adipose tissue, total WAT= epi-WAT+Per-WAT+Ing-wat.

To evaluate our hypothesis, we investigated the therapeutic potential of 4-HPA in alleviating obesity in HFD-fed mice. Mice on a HFD were administered 4-HPA (30 mg/kg BW, HFD4A) or normal saline via oral gavage daily for 16 weeks. At the end of the treatment period, we collected blood and tissues samples for analysis. Administration of 4-HPA resulted in approximately 0.5 μmol/L 4-HPA in the circulatory system, which produced similar levels of plasma 4-HPA under 300 mg/kg BW RSV treatment (Supplementary Figure S3A). 4-HPA treatment significantly mitigated obesity-related parameters compared with the HFD group, as evidenced by reductions in body weight, body weight gain, WAT weight and adipocyte size (Figure 5a-e). Notably, there was no significant difference in mean energy intake between the HFD and HFD4A groups (Figure 5d). Additionally, 4-HPA treatment improved dyslipidemia, evidenced by lower triglyceride (TG) and total cholesterol (TC) levels (Supplementary Figure S4A). Histological analysis revealed significant reductions in macrosteatosis, hepatocyte ballooning, and intrahepatic triglyceride accumulation in the livers of HFD-fed mice treated with 4-HPA (Supplementary Figure S4B). Furthermore, 4-HPA treatment enhanced glucose homeostasis and insulin sensitivity, as demonstrated by reduced fasting glucose and insulin levels, improved oral glucose tolerance test (OGTT) and insulin tolerance test (ITT) results, and decreased homeostasis model assessment of insulin resistance (HOMA-IR) (Figure 5g-m). Systemic inflammation was also notably reduced, with increased serum IL-10 levels and decreased levels of IL-1β, TNFα, IL-6, and LPS (Figure 5n-r).

**Figure 6.**
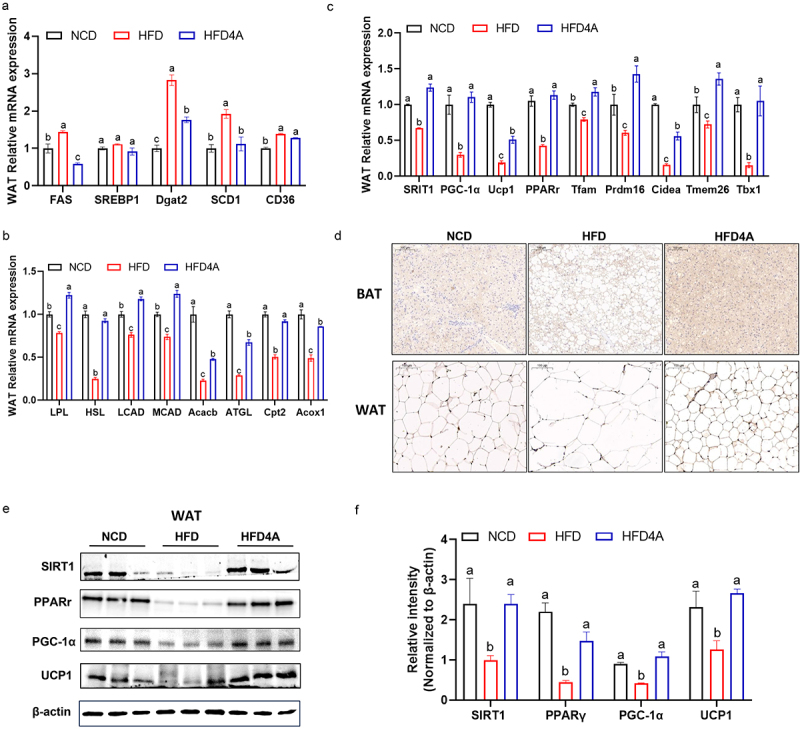
4-HPA treatment improves lipid metabolism in hfd-fed mice. (a) mRNA expression of fatty acid lipogenesis in WAT. (b) mRNA expression of fatty acid oxidation in WAT. (c) mRNA expression of thermogenesis in WAT. (d) UCP1 immunohistochemical staining in WAT. (e, f) Western blotting showed the expression of SIRT1 pathway-related proteins. Values are expressed as the mean±sem. Different letters (a, b and c) mean significant difference in different treatment groups (*p* < 0.05).

To further assess the impact of 4-HPA on lipid metabolism, we analyzed the mRNA expression of genes associated with lipogenesis, fatty acid oxidation, and browning in WAT. The expression levels of lipogenic genes, including FAS, Dgta2, and SCD1, were elevated in HFD-fed mice but were significantly reduced with 4-HPA treatment (Figure 6a). There were no significant differences in the expression of SREBP1 and CD36 among the groups. For fatty acid β-oxidation markers, 4-HPA treatment significantly upregulated the mRNA expression of LPL, HSL, LCAD, MCAD, Acacb, ATGL, Cpt2, and Acox1 in HFD-fed mice (Figure 6b). Additionally, genes associated with adipocyte browning, such as TFAM, PRDM16, CIDEA, TMEM26, and TBX1, which were downregulated in the HFD group, were markedly upregulated following 4-HPA treatment (Figure 6c). Collectively, these results indicated that 4-HPA treatment at a lower dose effectively reversed obesity and metabolic disorders in HFD-fed mice, which may have an analogues effect of RSV at a higher dose.

### 3.5. 4-HPA protect against obesity via the SIRT1 pathway

SIRT1 has been well-recognized as a protective molecule against obesity and related metabolic disorders. RSV, the precursor of 4-HPA, is known to activate SIRT1; however, *in vitro* studies have shown that RSV dose not directly activate SIRT1. Theis suggests RSV’s *in vivo* effects may involve an indirect mechanism for SIRT1 activation. Since microbial metabolites are absorbed directly through the gut epithelium into bloodstream, we hypothesized that these metabolites could influence SIRT1 activation. To investigate this hypothesis, we analyzed the mRNA and protein levels associated with the SIRT1 signaling pathway. We observed a significant increase in the expression of key SIRT1 pathway genes, including SIRT1, PGC-1a, PPARr and UCP1 (Figure 6c). Additionally, immunohistochemistry analysis revealed that 4-HPA treatment significantly upregulated UCP1 levels in WAT and BAT (Figure 6d). Western blot analysis confirmed elevated protein level of SIRT1, PGC-1α, PPARα and UCP1 in WAT (Figure 6e). These findings suggest that 4-HPA induces browning in WAT to attenuated obesity at least partially through the SIRT1 pathway.

**Figure 7.**
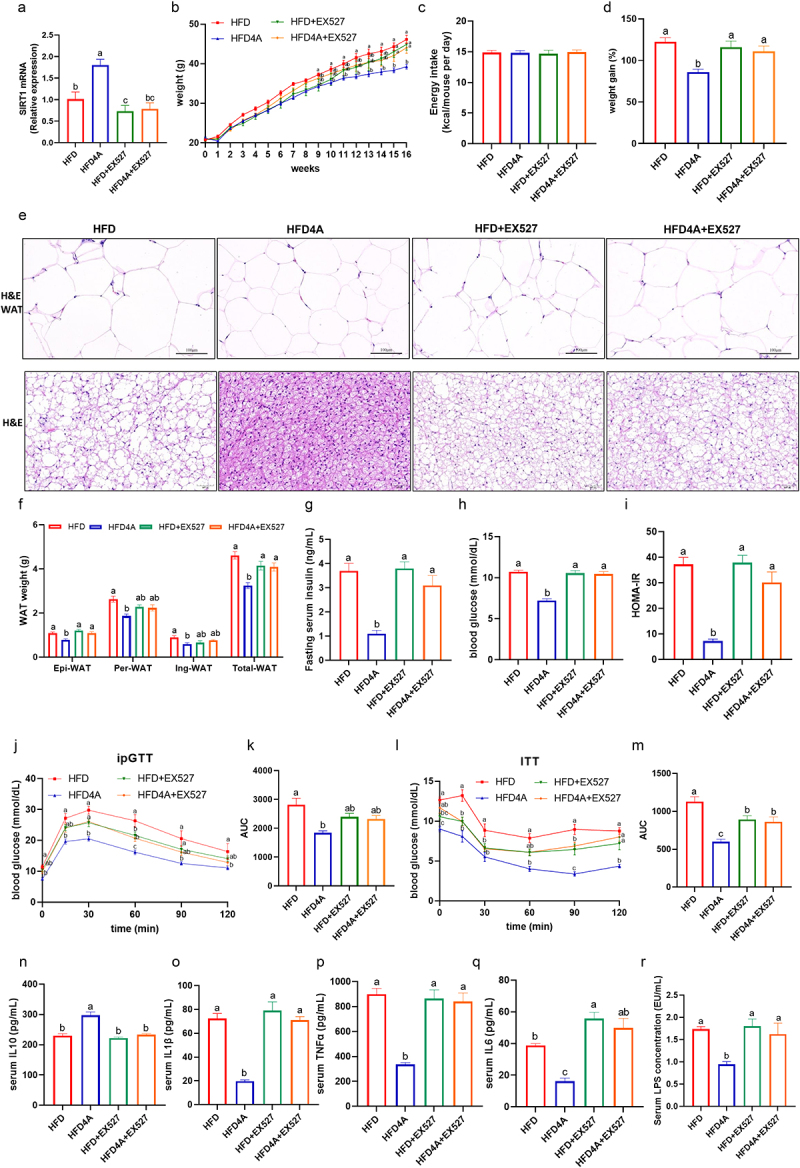
4-HPA protects against hfd-induced obesity through SIRT1 signaling pathway. mice were fed with HFD and HFD4A (30 mg/kg/day) with or without SIRT1 antagonist E×527(10 mg/kg/day) for 16 weeks. (a) The expression of SIRT1. (b) Body weight. (c) Body weight gain. (d) Energy intake. (e) H&E staining of white adipose and brown adipose tissue sections (scale: 100 μm); (f) WAT weight. (g) blood glucose. (h) Fasting serum insulin. (i) HOMA-ir. (j) Intraperitoneal glucose tolerance test (ipGTT) and (k) the calculated AUC. (l) Insulin tolerance test (ITT) and (m) the calculated AUC for ITT. (n) Serum IL-10 level. (o) Serum IL-1β level. (p) Serum tnf-α level. (q) Serum IL-6 level. (r) Serum LPS level. (S) The mRNA expression of lipid metabolism in WAT. Values are expressed as the mean±sem. Different letters (a, b and c) mean significant difference in different treatment groups (*p* < 0.05).

**Figure 8.**
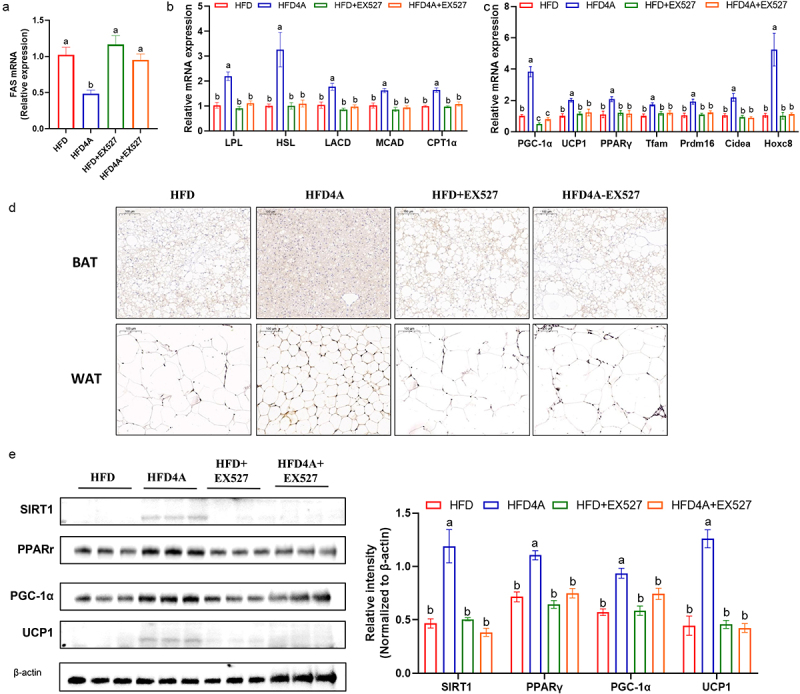
4-HPA regulates lipid metabolism in hfd-fed mice through SIRT1 signaling pathway. (a) mRNA expression of FAS in WAT. (b) mRNA expression of fatty acid oxidation in WAT. (c) mRNA expression of thermogenesis in WAT. (d) UCP1 immunohistochemical staining in WAT. (e, f) Western blotting showed the expression of SIRT1 pathway-related proteins. Values are expressed as the mean±sem. Different letters (a, b and c) mean significant difference in different treatment groups (*p* < 0.05).

To further validate whether the anti-obesity effects of 4-HPA depend on SRIT1 activation, we co-administered 4-HPA with the SIRT1-specific inhibitor E×527in HFD-fed mice. E×527significantly inhibited SIRT1 activity, and 4-HPA-mediated SIRT1 activation was completely abolished by E×527treatment (Figure 7a). Additionally, E×527treatment nullified the beneficial effects of 4-HPA on body weight, body weight gain, adipose size, glucose/insulin sensitivity and inflammatory cytokine levels (Figure 7b-r). We also assessed lipid metabolism markers in the WAT of these mice. The results indicated that 4-HPA’s ability to reduce lipogenic gene expression, such as FAS (Figure 8a), and to increase the expression of fatty acid β-oxidation genes (LPL, HSL, LACD, MACD and CPT1a) and browning genes (TFAM, PRDM16, CIDEA and HOXC8) was impaired by E×527treatment (Figure 8b, c). Additionally, the activation of the SIRT1 signaling pathway by 4-HPA, as evidenced by mRNA and protein levels of SIRT1, PPARr, PGC1α and UCP1 were also abolished by E×527(Figure 7a, 8c-e). These results collectively support the conclusion that 4-HPA exert its anti-obesity effects through a SIRT1-dependent pathway.

## 4. Discussion

Although previous studies have shown profound effects of dietary RSV interventions on anti-obesity for decades, mechanisms underlying these beneficial effects remain elusive.^16,25^ More and more attention has been made to contributions of gut bacterial toward metabolic homeostasis.^26,27^ Bringing gut microbiome and gut metabolomics into mechanistic understating of dietary RSV intervention will help us to resolve the puzzle of why those interventions target multiple organs with extremely low bioavailability.^17,27^ In this study, we found that dietary supplementation of RSV protected gut microbiota dysbiosis in HFD-fed mice, consistent with previous studies.^17^ Moreover, the anti-obesity effect of RSV is transferrable through FMT and dismission with antibiotic treatment. We identified for the first time that 4-HPA, a gut microbial metabolite of RSV, contributed to the protective effects of RSV against HFD-induced obesity by improving lipid metabolism and stimulating browning of WAT. The underlying mechanism for 4-HPA-induced anti-obesity effect was found to be associated with SIRT1-mediated signaling pathway. Thus, our results suggest that microbiota-4-HPA-SIRT1 axis could have tremendous potential for preventing and treating fat metabolism, like obesity.

Emerging evidences reported that gut microbiota play a causal role in the pathogenesis of obesity.^27,29^ Metabolic studies revealing high concentrations of RSV in the intestine following ingestion.^12^ These findings prompted us to investigate whether RSV exerts its anti-obesity effects through modulation of the gut microbiota. Our results shows that HFD feeding induced a dramatic shift in the gut microbiota of mice by increasing the proportion of *Firmicutes* and decreasing the proportion of *Bacteroidetes*. This diet-induced reshape in the microbial structure of HFD-fed mice is a typical characteristic of obesity-driven dysbiosis and in consistent with previous publications. Our studies further show that HFD leads to a dramatic enrichment of *Lactobacillus* up to about 50% and significantly decreased to the normal level after RSV treatment. Notably, it has been reported that the abundance of *Lactobacillus* was significantly increased in fecal samples of diabetic, which is similar to the changes observed in obesity.^21,30,31^ Consistently, Zhai et al demonstrated that oral gavage of *Lactobacillus* or its metabolites significantly increased intestinal lipid absorption, enhanced fatty acid uptake in WAT and induced fat mass accumulation.^32^ Moreover, target to inhibit intestinal *Lactobacillus* by a specific diet or antibiotics is likely to be beneficial to decrease lipid absorption and combat obesity. Hence, *Lactobacillus* of facultative anaerobic bacteria may be considered as the potential target for the “Bad” gut microbiota triggered by HFD induced obesity or diabetes. However, some *Lactobacillus* species have been recognized as probiotics to ameliorated inflammation, hyperlipidemia, hyperglycemia and the mucus layer thickness in HFD-fed mice.^33,34^ Furthermore, the abundance of the genus *Erysipelotrichaceae*, *Enterorhabdus (Coriobacteriaceae)* and *Lachnospiraceae_NK4A136_group* have been recurrently associated with host dyslipidemic phenotypes in mice and humans in the context of obesity, metabolic syndrome and hypercholesterolemia.^22-34^ Besides, RSV supplementation raised HFD-induced decrement in the *Akkermansia, UBA1819* and *Bacteroides*, which were possesses numerous functional impacts toward human health and play important role in the progress of obesity.^35–37^ We also observed that antibiotics treatment prevents the protective effects of RSV against obesity, whereas the transfer of RSV treated microbiota to HFD recipient rats shows transferable protective effect to obesity. These results indicate that gut microbiota exerts crucial effects on the therapeutic efficacy of RSV against obesity.

Accumulating evidence suggests that metabolites generated by the gut microbiota are key signaling molecules in microbial communities, as well as in host-microbial cross-talk.^38,39^ Consequently, defining and characterizing microbial-derived metabolites can aid in complementing microbial functions and deciphering host-microbiota interplay in health and disease.^40^ Our results showed that RSV administration with antibiotics could not alleviate obesity, indicating that bioactive metabolites produced by gut microbiome play an essential role in protecting against obesity.^41,42^ Aromatic phenolic acid is the crucial gut metabolite of polyphenols including RSV.^43^ Additionally, we observed a significant decrease in 4-HPA levels in mouse feces after bacterial clearance by antibiotics, suggesting a crucial contribution of the gut microbiota toward the homeostasis of 4-HPA in the host. However, the related research about the anti-obesity effect of 4-HPA are limited. Our previous study confirmed that at physiological concentrations (0.5–2 μmol/L), 4-HPA and 3-Hydroxyphenylpropionic acid significantly decreased lipogenesis gene expression and increased fatty acid oxidation gene expression *in vitro*.^17^ In the present study, we further to explore the anti-obesity function of 4-HPA administration *in vivo* and found that 4-HPA decrease the body weight, improve glucose and lipid metabolism hemostasis, and promoted the browning of subcutaneous WAT. Moreover, 4-HPA appears to induce the expression of many brown and beige phenotypes genes including CIDEA, TMEM26 and TBX1. Mitochondrial genes and transcriptional co-regulators, such as SIRT1, UCP1, PGC-1a, TFAM and PRDM16 were also significantly expressed in WAT of HFD4A group. Spearman’s correlation analysis indicated that the levels of the 4-HPA were negatively associated with all obesity traits (Supplementary Figure S2 G, H). The existing research on the potential of 4-HPA to improve obesity remains limited. Other studies exploring the metabolic benefits of 4-HPA can offer valuable insights into its functional roles, enhancing our understanding of its anti-obesity effects. Claesen et al., reported that 4-HPA is sufficient to reverse HFD-induced hepatic steatosis and liver injury.^43^ Similarly, treatment with 4-HPA ameliorated APAP-induced liver injury by increasing Nrf2 translocation to nucleus and enhancing the activity of antioxidant enzymes.^44^ Moreover, Li et al. reported that 4-HPA could improve alcoholic liver disease by modulating lipid-related gene expression such as PGC1α, FASN, ACACA1α and Dgtat1/2.^45^ Therefore, our observation suggests that gut metabolites, acting as a postbiotic, may be a novel strategy against HFD-induced obesity. Extensive clinical trials are required to further verify the pharmacological effects of 4-HPA on obesity.

Mechanistically, SIRT1 is an important regulator of metabolic processes such as lipolysis, fatty acid oxidation and mitochondrial activity, which has been already recognized as a clinical drug target.^46^ The activation of SIRT1 at WAT level occurs and leads to the modulation of PPARγ, that with PRDM16 and PGC-1a, promote transcription of genes specific of BAT.^8,47^ SIRT1 has been shown to be activated by RSV *in vivo*, but raised questions about the ability to activated SIRT1 *in vitro*.^13^ Based on the high concentration of RSV in intestine after administration as well as the physiological functions of gut metabolites which promoted us to speculate whether the gut microbiota metabolites ameliorated obesity by activating SIRT1.^48^ Our study revealed that the impact of RSV on activating SIRT1 was deleted in antibiotic-treated mice, yet transplanting the microbiota obtained from RSV-treated mice into recipient mice upregulated the expression of SIRT1. Additionally, we found that 4-HPA treatment induced the gene and protein expression of SIRT1 signaling pathway in WAT and BAT, such as SIRT1, PGC-1a, PPARγ and UCP1. However, 4-HPA failed to exhibit additional beneficial effects on obesity when these mice were cotreated with E×527(a selective sirtuin inhibitor), suggesting that the protective effects of 4-HPA on HFD-induced obesity were associated with the activation of SIRT1. Moreover, E×527treatment block the effect of 4-HPA on SIRT1 signaling activation, including the expression of UCP1, PPARγ and PGC-1a in WAT and BAT, suggesting that UCP1, PGC1a and PPARγ levels might be strongly correlated with the expression of the SIRT1 signaling.

In summary, our results demonstrated that RSV could significantly relieve HFD-induced obesity and inflammation in a gut microbiota-dependent manner. RSV was found to improve microbial imbalance and significantly enrich gut microbiota-derived metabolites (4-HPA). Supplementation with 4-HPA could reduce obesity-related symptoms and inflammation in HFD-fed mice by activating SIRT1 to modulate adipose tissue browning and thermogenesis. Our findings highlight the significance of the gut microbiota-derived 4-HPA-SIRT1 axis in ameliorated obesity and suggest 4-HPA as a postbiotic that is liberated from RSV by gut microbiota, offering promising therapeutic approaches for obesity and related metabolic diseases.

## Supplementary Material

Supplemental Material

## Data Availability

The data that support the findings of this study are available in the article or from the corresponding author upon reasonable request.

## Author statement

Pan Wang and Xiaoyan Zhao conceived the idea and designed this study. Pan Wang, Ruiqi Wang, Yuanyuan Zhao, Wenting Zhao, Dan Wang and Zhiwen Ge acquired experimental data. Yue Ma provided material support. Pan Wang drafted the manuscript and obtaining funding. All authors contributed to the article and approved the submitted version.
