# Supplementary material for: Gut microbiota-derived 4-hydroxyphenylacetic acid from resveratrol supplementation prevents obesity through SIRT1 signaling activation
Source: Gut Microbes. 2024 Dec 26;17(1):2446391. doi: 10.1080/19490976.2024.2446391 (PMC12931687; doi:10.1080/19490976.2024.2446391)
Supplement: Supplemental Material [file KGMI_A_2446391_SM0731.zip › supplementary_Figure_total.docx]

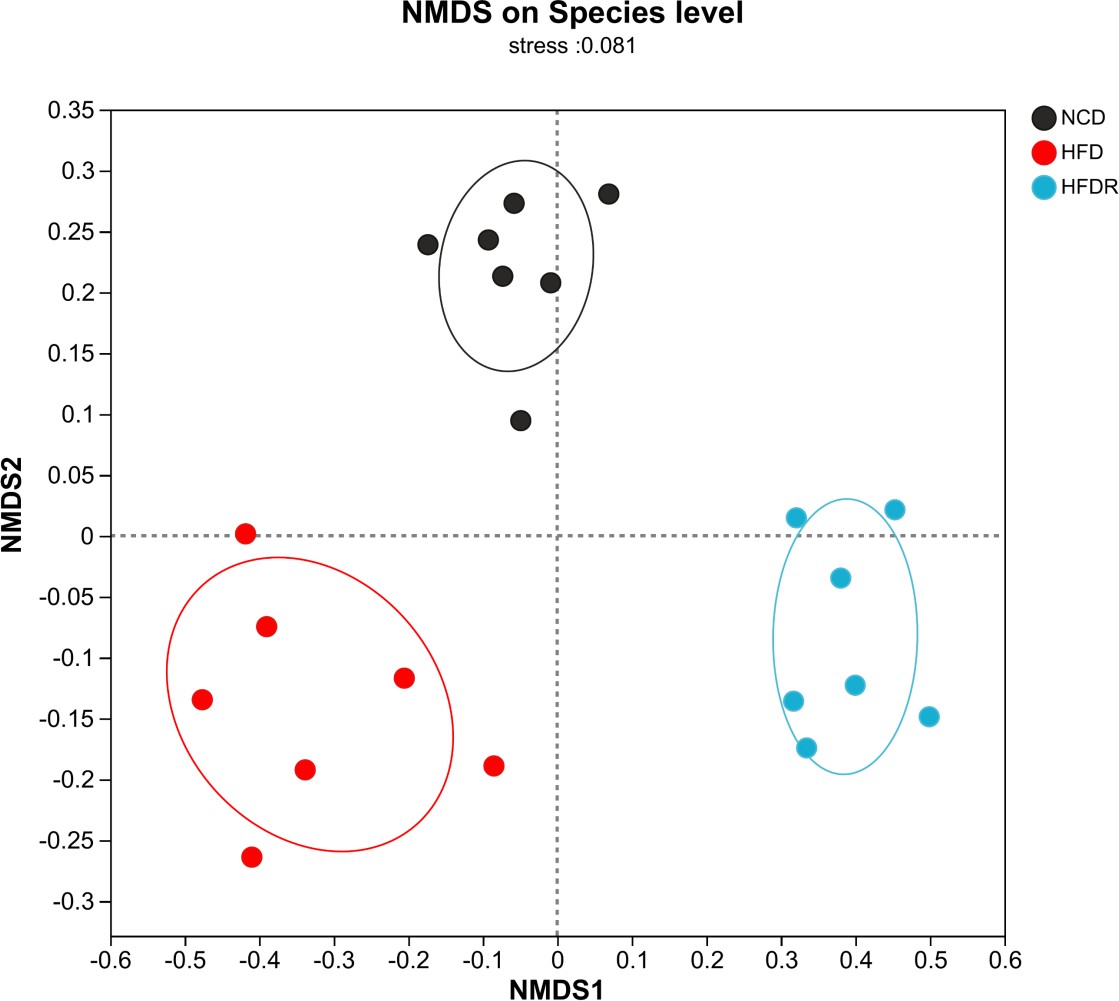
A


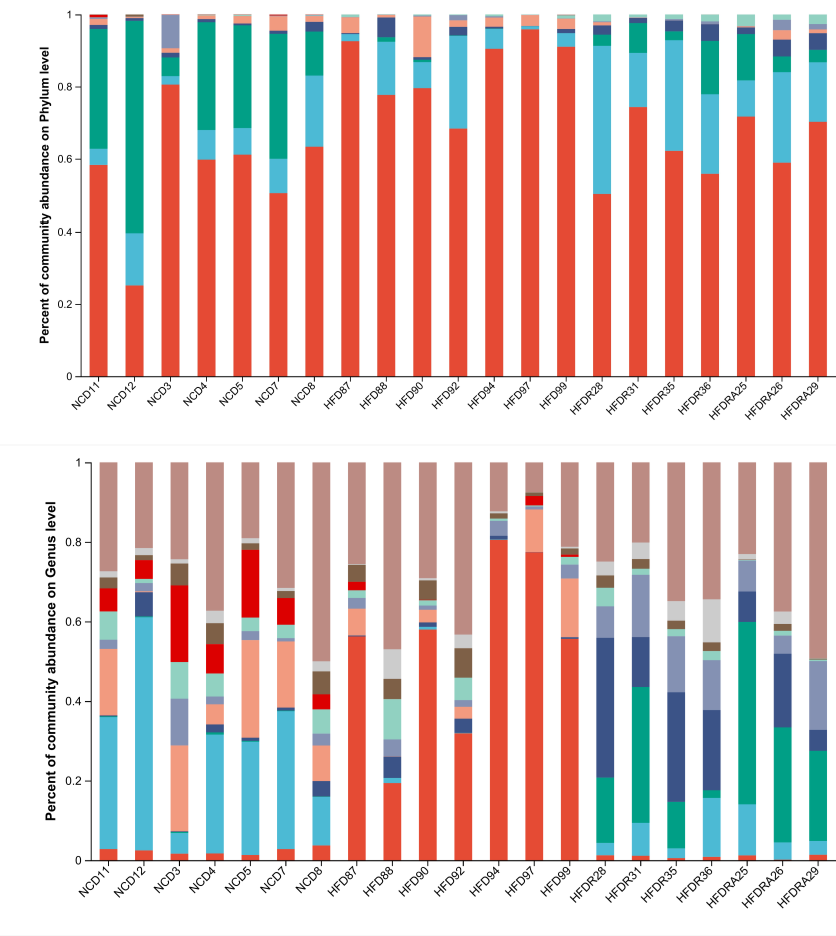

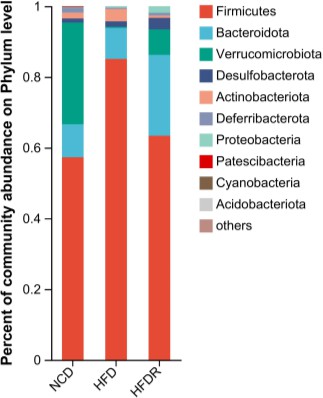
B

C


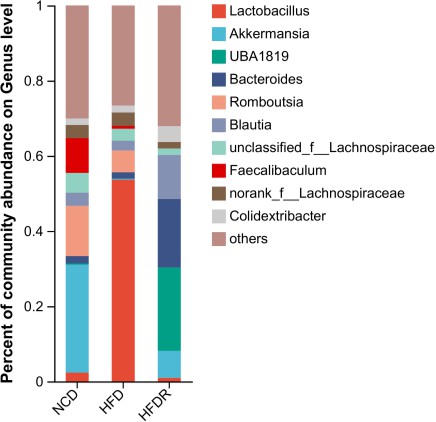


# Supplementary Figure 1. Effect of RSV on gut microbiota composition in HFD-fed mice.

(A) The difference in gut microbiota structure in NCD-, HFD-, HFDR-fed mice assessed by NMDS; (B) Gut bacterial composition at the phylum level in the three groups; (B) Gut bacterial composition at the genus level in the three groups (n=7).

A C E


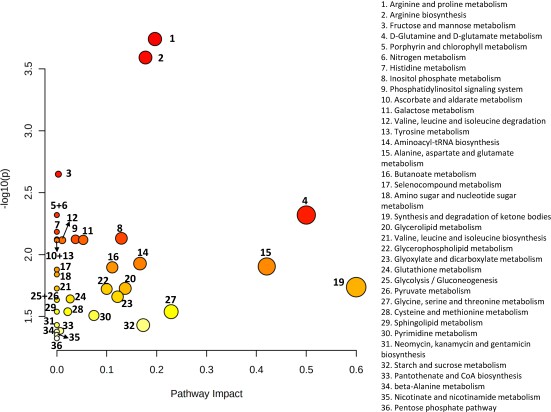

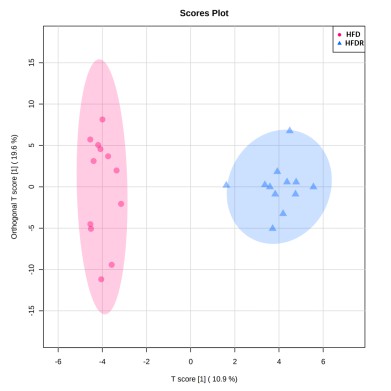


D

F


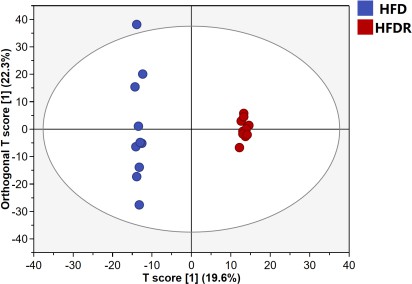

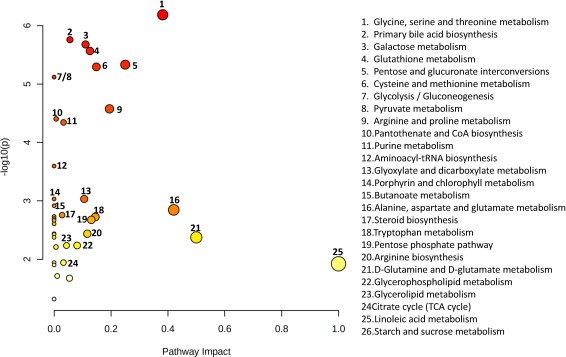


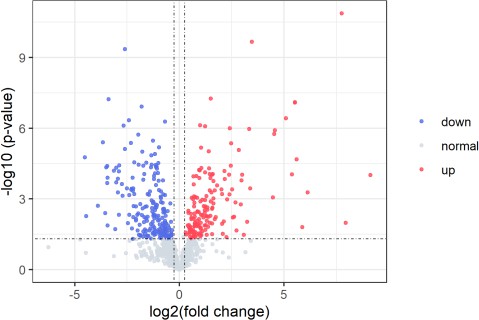


B


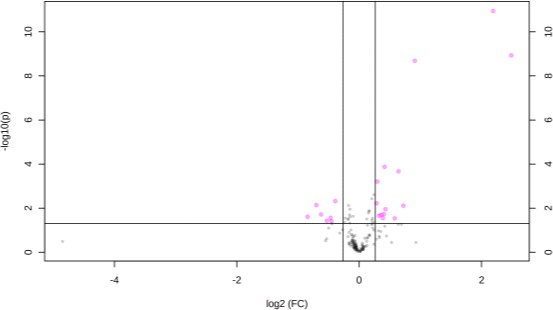


G H


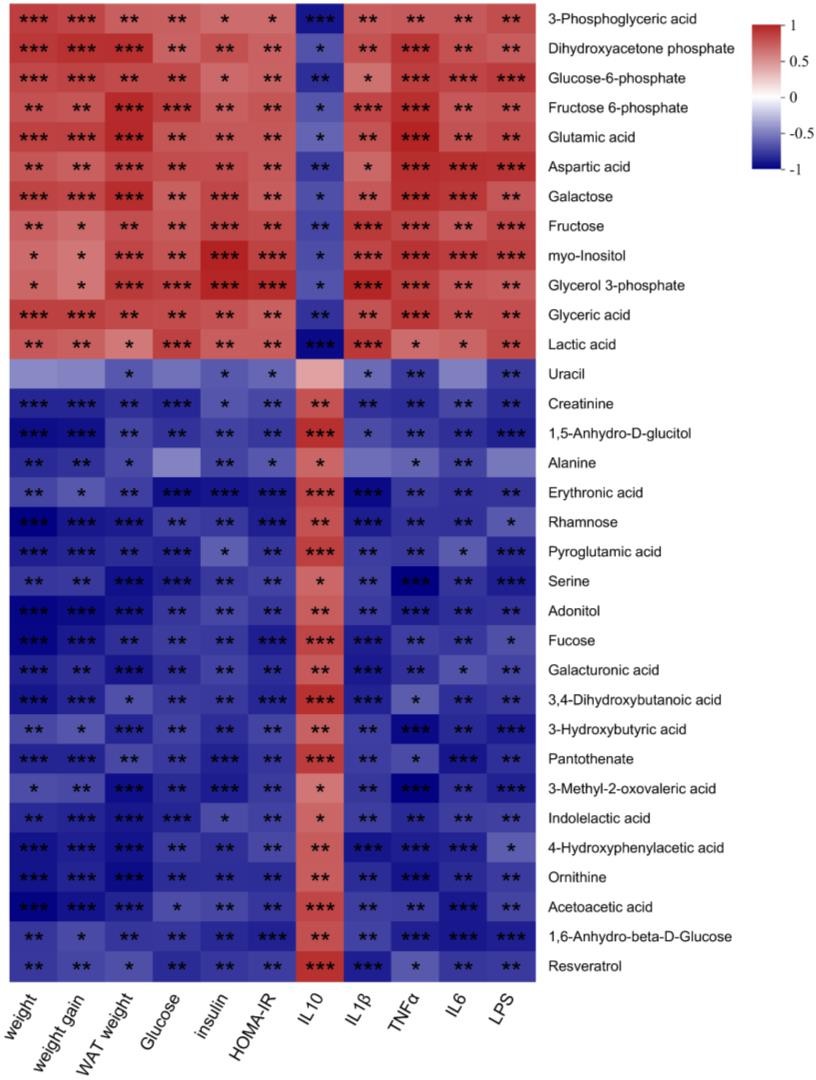

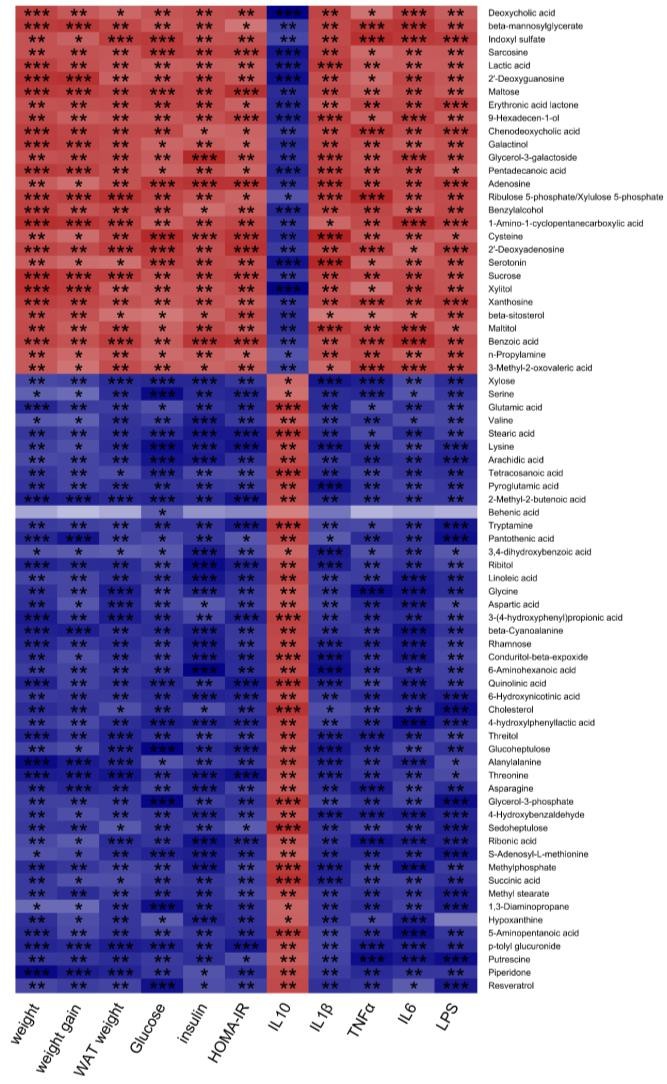


**Supplementary Figure 2. GC-MS metabolomics analysis conducted for microbiome and serum of HFD and HFDR groups.** (A, B) Volcano plots of statistically significant other common metabolites compared with HFD and HFDR groups in fecal (n=10) and serum (n=12). The x-axis is the log2-fold change, and the y-axis is the log10-p value; (C, D) Partial least-squares discriminant analysis (PLS-DA) of metabolomic profiles in fecal and serum of HFD and HFDR groups; (E, F) Pathway enrichment analysis of the significantly altered metabolites in fecal and serum of HFD and HFDR groups. The x-axis represents the pathway impact, and the y-axis represents the pathway enrichment. (G, H) Correlations between metabolites and obesity-related symptoms with P values < 0.05 were considered statistically significant.

A


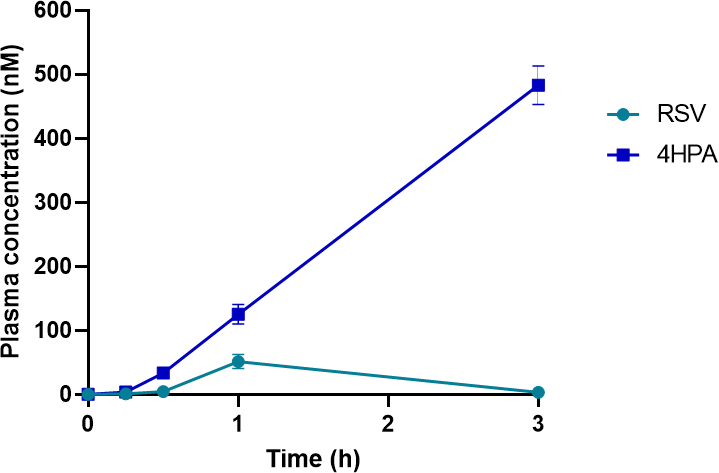


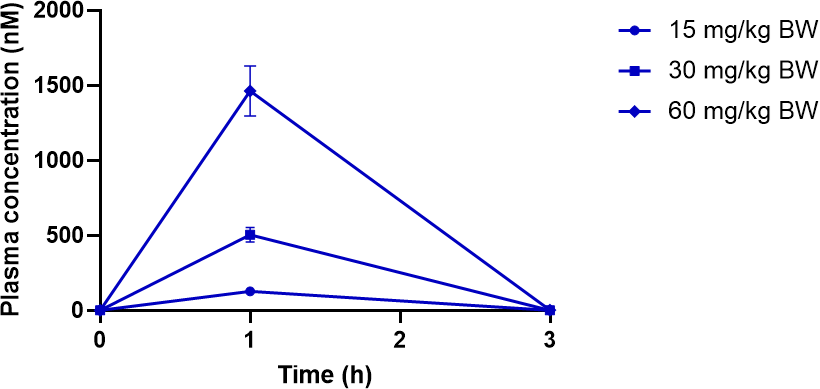
B

**Supplementary Figure 3.** (A) The plasma concentration of RSV and 4-HPA were measured before 0.25, 0.5, 1 or 3 hours after RSV(300 mg/kg BW) administration in mice. (B) The plasma concentration of PCA after oral gavage of PCA (15, 30 or 60 mg/kg BW). The results are the means±SEM, n=3.


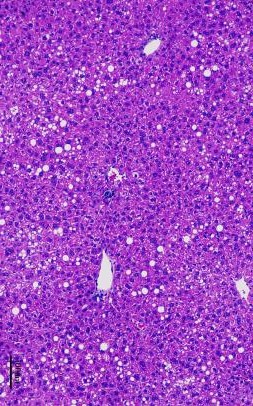


# NCD HFD HFD4A


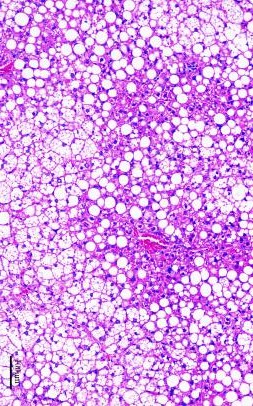

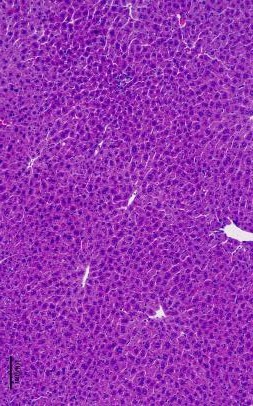


**
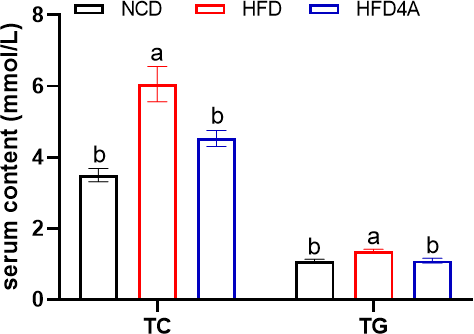
**

**Supplementary Figure 4. 4-HPA attenuated HFD-induced liver steatosis.** (A) Serum concentration of TC and TG; (B) H&E staining in liver (scale: 100 μm)
