# Supplementary material for: Gut microbiota-derived 4-hydroxyphenylacetic acid from resveratrol supplementation prevents obesity through SIRT1 signaling activation
Source: Gut Microbes. 2024 Dec 26;17(1):2446391. doi: 10.1080/19490976.2024.2446391 (PMC12931687; doi:10.1080/19490976.2024.2446391)
Supplement: Supplemental Material [file KGMI_A_2446391_SM0731.zip › supplementary method.docx]

**Supplementary Methods**

**16S rRNA amplicon sequencing**

Genomic DNA was extracted from each fecal sample using the QIAamp-DNA stool mini kit (Qiagen, Hilden, Germany). The integrity of the extracted DNA was examined by electrophoresis in 1% (w/v) agarose gels. The V3-V4 hyper-variable regions of the 16S rRNA gene was amplified using the primers 338F (5’-GTGCCAGCMGCCGCGG-3’) and 806R (5’-CCGTCAATTCMTTTRAGTTT-3’) [2]. PCR amplification was performed on an ABI GeneAmp®9700 PCR instrument (AppliedBiosystems, Foster City, CA, USA) and the products were quantified with a QuantiFluorTM-ST Handheld Fluorometer with UV/Blue Channels (Promega Corporation, Madison, WI, USA). PCR reactions were carried out in 20 μL reactions with TransGen AP221-02 kit (Beijing, China); 0.8μM of forward and reverse primers,

and about 10 ng template DNA. Thermal cycling consisted of initial denaturation at 95 °C for 3 min, followed by 30 cycles of denaturation at 95 °C for 30 s, annealing at 55 °C for 30 s, and elongation at 72 °C for 45 s. Finally, 72 °C for 10 min. Sequencing and processing of the PCR amplicons was performed on an Illumina Miseq platform by Majorbio Bio-Pharm Technology Co., Ltd. (Shanghai, China).

Reads with average PHRED qualities below 20 across a 50 bp sliding window, with final lengths shorter than 50 bp, and reads containing ambiguous bases were filtered out. Merged amplicons were obtained from reads overlapping at least 10 bp with a maximum error rate of 0.2 with FLASH (Magoc & Salzburg), and barcodes were trimmed with Trimmomatic v0.33 [3]. Good reads were dereplicated and singletons removed before clustering at 97% identity into operational taxonomic units (OTUs) and chimera-checked with USEARCH v7.0 [4]. An abundance matrix was then constructed by mapping the good reads to the OTUs’ representative sequences. Finally, representative sequences were classified using RDP against the SILVA (SSU138) database [5].

Analyses for rarefaction curves, and calculation of α-diversity (Shannon index, Simpson index and Shannoneven index) were performed using the MOTHUR program [6]. Compositional differences between samples were analyzed by calculating unweighted-UniFrac and weighted-UniFrac distances and visualizing the dissimilarity matrix with principal coordinate analysis (PCoA). The bar plot was implemented by R package (R4.2.2). To identify bacterial taxa that contributed to the significant differences across all groups, we applied linear discriminant analysis effect size (LEfSe) based on the Kruskal–Wallis H test followed by linear discriminant analysis (LDA) to measure the effect size of each abundant taxon applying two filters (P < 0.05 and LDA score > 3.5) [7].

Supplementary references

[1] J. DeFuria, G. Bennett, K.J. Strissel, J.W. Perfield, 2nd, P.E. Milbury, A.S. Greenberg, M.S. Obin, Dietary blueberry attenuates whole-body insulin resistance in high fat-fed mice by reducing adipocyte death and its inflammatory sequelae, J Nutr 139(8) (2009) 1510-6.

[2] S. Wang, M. Huang, X. You, J. Zhao, L. Chen, L. Wang, Y. Luo, Y. Chen, Gut

microbiota mediates the anti-obesity effect of calorie restriction in mice, Sci Rep 8(1) (2018) 13037.

[3] A.M. Bolger, M. Lohse, B. Usadel, Trimmomatic: a flexible trimmer for Illumina sequence data, Bioinformatics 30(15) (2014) 2114-2120.

[4] R.C. Edgar, Search and clustering orders of magnitude faster than BLAST, Bioinformatics 26(19) (2010) 2460-1.

[5] C. Quast, E. Pruesse, P. Yilmaz, J. Gerken, T. Schweer, P. Yarza, J. Peplies, F.O. Glöckner, The SILVA ribosomal RNA gene database project: improved data processing and web-based tools, Nucleic Acids Res 41(Database issue) (2013) D590-6.

[6] P.D. Schloss, S.L. Westcott, T. Ryabin, J.R. Hall, M. Hartmann, E.B. Hollister, R.A. Lesniewski, B.B. Oakley, D.H. Parks, C.J. Robinson, J.W. Sahl, B. Stres, G.G. Thallinger, D.J. Van Horn, C.F. Weber, Introducing mothur: open-source, platform-independent, community-supported software for describing and comparing microbial communities, Appl Environ Microbiol 75(23) (2009) 7537-41.

[7] N. Segata, J. Izard, L. Waldron, D. Gevers, L. Miropolsky, W.S. Garrett, C. Huttenhower, Metagenomic biomarker discovery and explanation, Genome Biol 12(6) (2011) R60.
